# 218. Attenuated PreFusion F Antibody Response to RSV Vaccines in Solid Organ Transplant Recipients

**DOI:** 10.1093/ofid/ofae631.076

**Published:** 2025-01-29

**Authors:** Andrew H Karaba, Camille Hage, Isabella Sengsouk, Aura Abdeon, Woudase Gallo, Xori Green, Maggie Chahoud, Aaron Tobian, William Werbel

**Affiliations:** Johns Hopkins University, Baltimore, MD; Johns Hopkins University, Baltimore, MD; Johns Hopkins University, Baltimore, MD; Johns Hopkins University, Baltimore, MD; Johns Hopkins University, Baltimore, MD; Johns Hopkins University, Baltimore, MD; Johns Hopkins University, Baltimore, MD; Johns Hopkins University, Baltimore, MD; Johns Hopkins, Baltimore, Maryland

## Abstract

**Background:**

RSV causes serious morbidity in solid organ transplant recipients (SOTRs). Novel RSV vaccines are highly protective in the general population, yet immunogenicity among SOTRs is unknown.

Demographic and Transplant Characteristics of SOTRs Reporting RSV Vaccination

**Figure:**
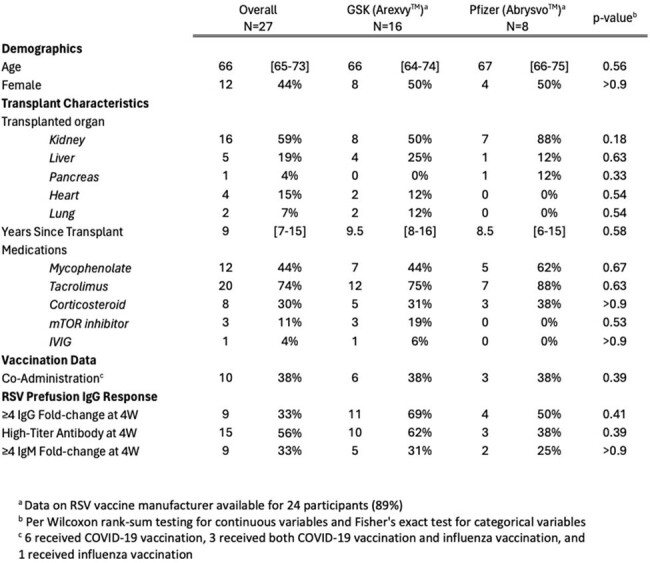

**Methods:**

Within a national, prospective cohort study SOTRs submitted whole blood samples at baseline and 2, 4, and 12 weeks post receipt of RSV vaccination (RSVPreF3 [GSK, AREXVY™] or RSVpreF [Pfizer, ABRYSVO™]) between October 2023-March 2024. Plasma prefusion F IgG and IgM (preF Ab) was tested using the Meso Scale Discovery platform and compared between baseline and 4 weeks to assess fold-change (FC); the proportion achieving high-titer Ab (IgG ≥high-titer reference plasma [BEI Resources]) at 4 weeks was also assessed. Ab measures were compared between GSK and Pfizer vaccinees.

Change in Prefusion F IgG Titers following RSV Vaccination in SOTRs

**Figure:**
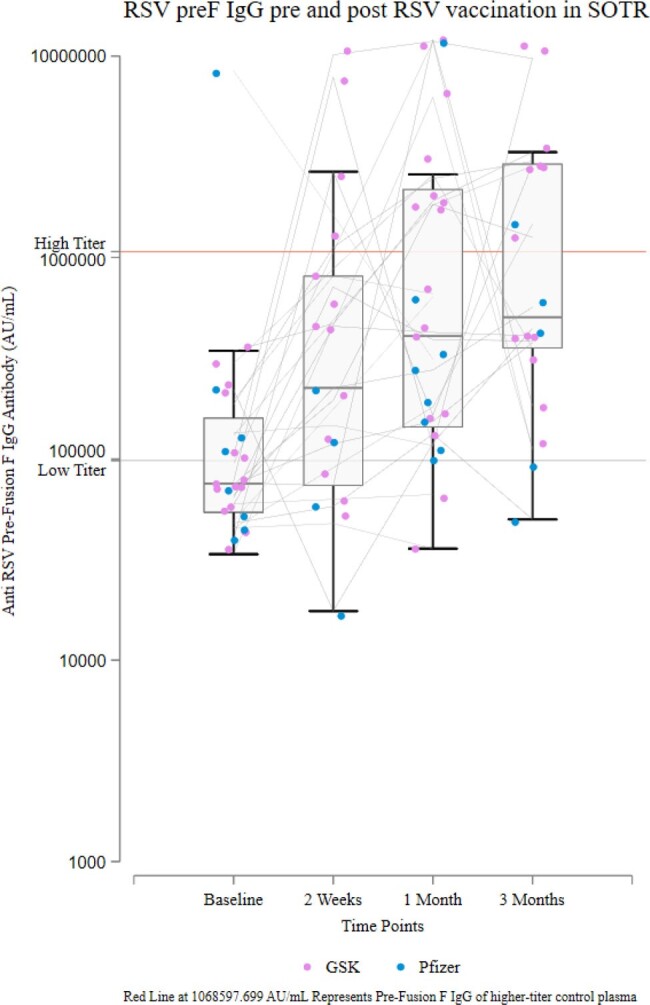

Blue dots represent Pfizer vaccine recipients and pink dots represent GSK vaccine recipients. Individual titer trajectories are connected by grey lines. High-titer response is denoted by the upper red horizontal line, corresponding to high-titer pooled control plasma.

**Results:**

Among 27 participants (median [IQR] age 65-73 years, 44% female, 59% GSK and 30% Pfizer vaccinees [11% unspecified]), median [IQR] preF IgG rose from 76,252 [59,116-185,845] to 424,618 [153,503-1,905,705] AU/mL by 4 weeks (median [IQR] FC 5.54 [2.06-13.26]). Overall, 67% demonstrated IgG FC ≥4 at 4 weeks (69% GSK vs 50% Pfizer vaccinees) and 52% demonstrated high-titer Ab at 4 week (62% GSK vs 25% Pfizer vaccinees); clinical and transplant factors were similar between response groups. By 12 weeks, 56% showed high-titer Ab (62% GSK vs 38% Pfizer vaccinees) (Figure). Response patterns varied, including greater IgM change from baseline to 4 weeks in GSK vaccinees (IgM FC ≥4: 31% GSK vs 25% Pfizer) and more pronounced IgG peak-and-wane in GSK vaccinees (median FC from 4 to 12 weeks 1.01 GSK vs 0.49 Pfizer). No participants reported RSV infection during follow up.

Prefusion F IgG Titers pre and post RSV Vaccination in SOTRs, by Vaccine Type

**Figure:**
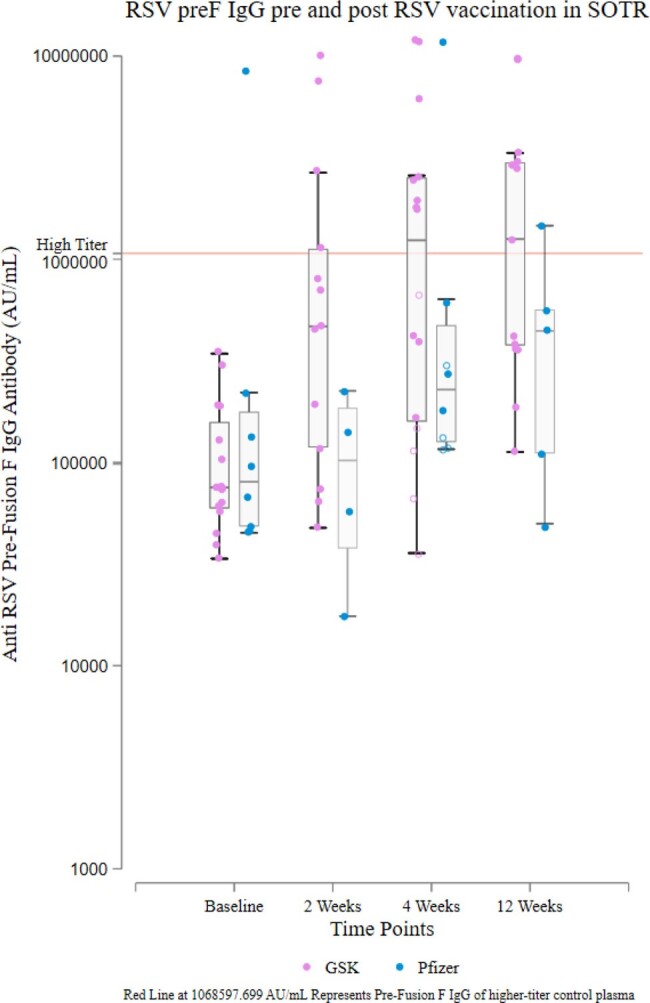

Blue dots represent Pfizer vaccine recipients and pink dots represent GSK vaccine recipients. High-titer response is denoted by the upper red horizontal line, corresponding to high-titer pooled control plasma. Open circles represent ≥ 4-fold change in titer at 4 weeks.

**Conclusion:**

RSV preF Ab responses are highly variable and often attenuated in SOTRs following RSV vaccination; 44% did not show high-titer Ab at 4 weeks. Response level and pattern vary by vaccine platform (i.e., adjuvanted versus unadjuvanted), which may imply different risk periods post vaccination and potential role for additional vaccine doses to improve immunoprotection.

Prefusion F IgM Titers pre and post RSV Vaccination in SOTRs, by Vaccine Type

**Figure:**
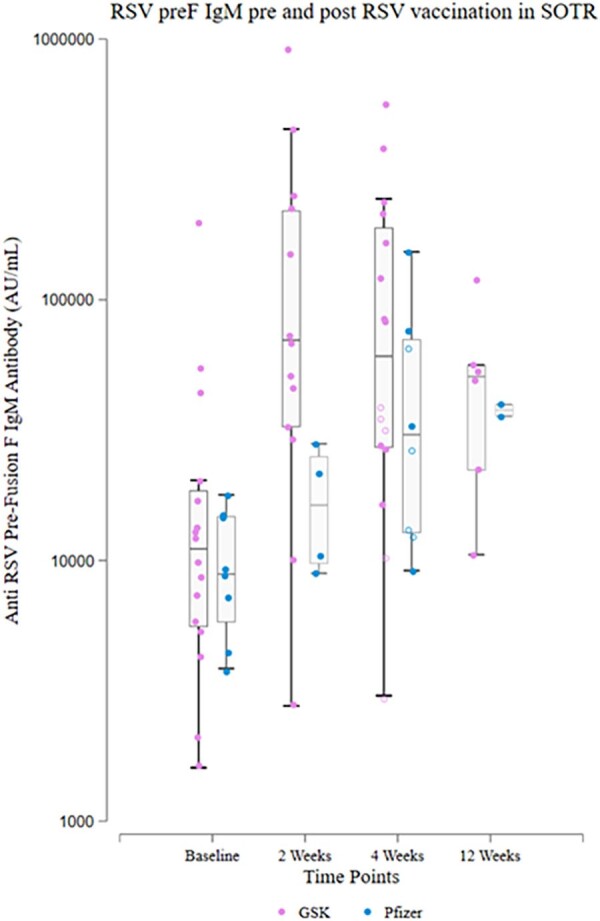

Blue dots represent Pfizer vaccine recipients and pink dots represent GSK vaccine recipients. Open circles represent ≥ 4-fold change in titer at 4 weeks.

**Disclosures:**

**Andrew H. Karaba, MD PhD**, Hologic: Advisor/Consultant **William Werbel, MD**, AstraZeneca: Advisor/Consultant|AstraZeneca: Honoraria|IDSA: Advisor/Consultant|Novavax: Advisor/Consultant

